# Construction and Characterization of an Attenuated Vaccine Vector Based on Cucumber Mosaic Virus RNA2

**DOI:** 10.3390/life16091489

**Published:** 2026-09-05

**Authors:** Zhao Wang, Shanshan Liu, Mingjing Zhu, Jiagan Zhang, Xueyuan Wang, Zhifei Liu, Kaiqiang Hao, Shuyuan Tian, Xuefeng Yuan, Chengming Yu

**Affiliations:** 1 Department of Plant Pathology, College of Plant Protection, Shandong Agricultural University, Tai’an 271000, China; w19853405001@163.com (Z.W.);; 2 Pest Integrated Management Key Laboratory of China Tobacco, Tobacco Research Institute, Chinese Academy of Agricultural Sciences, Qingdao 266101, China

**Keywords:** cucumber mosaic virus, RNA2, attenuated vaccines, cross protection

## Abstract

Plant viruses are major plant pathogens and account for nearly half of emerging plant diseases worldwide. To date, few effective management measures are available for the efficient control of plant viral diseases. Cross-protection based on attenuated vaccine is an effective strategy to prevent plant viral diseases. The primary requirement for the development of attenuated vaccines is a vector with excellent characteristics, such as low pathogenicity, genetic stability, and the capacity for stable insertion of exogenous fragments. In this study, the cucumber mosaic virus (Fny strain) was genetically modified to serve as an attenuated vaccine vector. Based on pre-termination of the 2b protein-coding region and deletion of the 3′ UTR in CMV RNA2, six RNA2 mutants, designated R2-2bPTI, R2-2bPTII, R2-2bPTIII, R2-2bPTIV, R2-2bPTV, and R2-2bPTVI, were constructed. Experiments with different lengths tobacco phytoene desaturase (PDS) fragment insertion evaluated the capacity of each mutant to accommodate exogenous fragment. Based on the R2-2bPTIV, a viral fragment insertion mutant R2-2bPTIV-TMPYTVPX targeting cucumber mosaic virus (CMV), tobacco mosaic virus (TMV), potato virus Y (PVY), tobacco vein banding mosaic virus (TVBMV), and potato virus X (PVX) was constructed. This mutant provided effective cross-protection against these targeted virulent viruses. This study developed a series of CMV-based vaccine vector, providing materials and data for the development of plant viral diseases attenuated vaccines.

## 1. Introduction

Plant viruses infect a wide range of hosts, including food and oil crops, Chinese medicinal herbs, and ornamental plants, causing approximately $60 billion of economic loss worldwide annually [1,2,3,4,5,6]. Plant virus could be spread through various ways including agricultural operations, insect vectors, and seeds [7]. Animals have evolved an adaptive immune system composed of specialized immune cells (T lymphocytes and B lymphocytes) and a circulatory system, which can generate long-lasting memory, however, plants do not have such a system and rely on the innate immunity of each cell, and thus plant viral diseases are uncontrolled upon occurrence [8,9,10,11].

Development of the effective prevention and control of plant virus diseases have long been a challenging and hot topic in the fields of plant pathology and virology [12].

The use of the cross protection mechanism of attenuated vaccines is an effective strategy for the prevention and control of plant viral diseases, with the advantages of long period, green and safe, and environmental friendliness. Two strains of pepino mosaic virus (PepMV), CH2 and VX1 were approved as plant virus vaccines by the European Union in 2015 and 2017 respectively. Which with no notable safety risks to date fully confirms the reliability and application potential of cross-protection based vaccines in plant viral disease management [13,14].

The cross protection of attenuated vaccines is that the plant could partial or complete resistant to a plant to infection of a severe strain of a virus after the intentional inoculation of a mild strain of the same virus [15,16]. A qualified attenuated vaccine must possess the following requirements: (1) systemic infection with inoculated plants, (2) mild symptoms or asymptomatic, (3) limited unintentional spread, (4) against a wide range of wild-type isolates, (5) effective control and ease of application, (6) genetic stability, (7) Does not synergistically harm the host with other viruses [17,18].

The mechanism underlying cross-protection comprises three major hypotheses: the coat-protein-mediated mechanism, RNA silencing (RNAi), and superinfection exclusion (SIE). It is widely accepted that the mechanism of cross protection is based on gene silence. During replication, attenuated vaccines produce small interfering RNAs (siRNAs) that can cleave the target mRNA, thereby inhibiting the replication of virus [16]. Many successful research and applications of cross protection based attenuated vaccines have been reported. For example, the attenuated strain 425 of alfalfa mosaic virus (AMV) has significant cross protection on the virulent strain AMV-T6 [19]. The attenuated vaccine strains of citrus tristeza virus (CTV) has effectively cross protection on the virulent strain CTV and used for controlling the harm of CTV [20,21]. The mixed attenuated strain V10 (VX1 + VC1) of Pepino mosaic virus (PepMV) can attenuate the symptoms of leaf wrinkling, necrosis, and spotted fruit caused by the strong strain of PepMV mixture (EU+CH) on tomatoes [22]. The Potato virus X RNA dependent RNA polymerase mutants E46A and E1001A could cross protect against severe infection of Potato virus X in both *Nicotiana benthamiana* and tomato [23]. On cucumber, melon, and zucchini, the attenuated strain ZYMV-WK of zucchini yellow mosaic virus (ZYMV) has a significant cross protection function against the severe type of ZYMV isolated from Connecticut, Florida, France, and Taiwan [24].

Current attenuated vaccines are derived from natural mutants and can provide cross protection only against a single virus. Viruses are diversity and rapidly evolution [25,26]. The co-infection of one host with multiple viruses is a common phenomenon that often occurs in nature [27]. It is necessary to rapidly generate the attenuated mutant that can provide cross-protection against multiple viruses simultaneously. The use of new molecular techniques opens up the possibility of identifying and understanding the mechanisms of the different natural enemies of plant pathogens and pest [28]. Using molecular biological methods to modify plant viruses by inserting gene fragments of other viruses to construct viral mutants that can produce the target siRNAs to inhibit viral replication is an effective strategy [29,30]. This approach not only allows for the precise manipulation of target virus genetic fragment to the vaccine vector but also enables the incorporation of multiple target viral gene sequences into a single viral vector quickly. This strategy offers a more efficient and versatile solution for the challenges posed by the diversity and rapid evolution of plant viruses. A suitable vaccine vector is the primary prerequisite for the successful implementation of this strategy.

Cucumber mosaic virus (CMV), the representative species of the genus *Cucumovirus* in the family *Bromoviridae* [31], and can be transmitted through seeds and over 80 species of aphids, infecting 1200 species in over 100 families. CMV infection causes symptoms such as leaf yellowing, malformations, and plant dwarfism, severely impacting yield and quality [32]. CMV is one of the plant viruses with the most diverse host range, the widest distribution, and the greatest economic impact. CMV was selected as one of the top 10 most scientifically/economically important plant viruses [33,34].

The CMV genome consists of three single-stranded positive-sense RNAs (RNA1, RNA2, and RNA3) and two subgenomic RNAs (RNA4A and RNA4), with a cap at the 5′ end and no poly(A) at the 3′ end. Each of the five viral RNAs encodes distinct viral proteins: 1a, 2a, 3a (MP), 2b, and coat protein (CP) [35]. The 1a protein encoded by RNA1 is essential for viral genome replication and influences viral pathogenicity [36,37]. The 2a and 2b proteins are encoded by RNA2. The 2a protein possesses RNA-dependent RNA polymerase (RdRp) activity and participates in the viral replication with the 1a protein [37,38]. The 2b protein encoded by RNA4A is responsible for viral movement, systemic symptoms expression, and suppression of host gene silencing [39,40]. RNA3 encodes the 3a protein (MP), which mediates cell-to-cell movement [41]. Coat protein (CP), encoded by RNA4, is involved in the encapsulation of genomic RNAs, aphid transmission, and the antiviral signaling pathway [42,43,44,45].

The characteristics of a broad host range, abundant isolates, and a tripartite genome structure provide multiple choices for the selection of mutation sites and the insertion of exogenous fragments. Thus, CMV constitutes a promising candidate for constructing viral vectors for developing attenuated vaccines [32,46,47]. In this study, six RNA2 mutants with 2b pre-termination, deletion of part of 2b and part of 3′-UTR of RNA2 were constructed using the CMV_Fny_ isolate. Pathogenicity, stability, cross-protection, and the maximum insert length of exogenous fragments were analyzed to evaluate the ability of attenuated vaccine vectors.

## 2. Materials and Methods

Infective cloning plasmids containing cDNA of CMV_Fny_ full-length genome RNAs were kindly donated by Prof. Xiaorong Tao from Nanjing Agricultural University [48]. Infective cloning plasmids containing cDNA of PVY(X97895), PVX(EU571480) full-length genome RNAs, *N. benthamiana* leaves infected with TMV(AF395129), TVBMV(EU734432) respectively were kindly donated by Prof. Xiangdong Li from Shandong Academy of Agricultural Sciences.

### 2.1. Plasmid Construction

Using the CMV_Fny_ RNA2 infectious cloning plasmid as the template, linearized mutants with restriction enzyme cleavage sites were obtained through site-directed mutagenesis PCR. These mutants were then digested with *Bam*HI (Takara, Beijing, China) and ligated with T4 DNA ligase (Takara) to form the self-ligated mutants, generating six CMV RNA2 mutants. Different-length fragments of *N. benthamiana* PDS coding region with *Bam*HI and *Sma*I were cloned, then cloned fragments and CMV RNA2 mutants were both digested with *Bam*HI and *Sma*I (Takara), and ligated with T4 DNA ligase, generating different-length PDS fragment insertion mutants based on CMV RNA2 mutants. 121 bp TMV conserved fragment, 100 bp PVY conserved fragment, 100 bp TVBMV conserved fragment and 100 bp PVX conserved fragment were cloned and overlapped in order of TMV, PVY, TVBMV, PVX via overlap PCR, then overlapped fragment and R2-2bPTIV were both digested with *Bam*HI and *Sma*I, and ligated with T4 DNA ligase, generating viral fragment insertion mutant R2-2bPTIV-T_M_P_Y_T_V_P_X_. All mutants were confirmed by PCR and sequencing (Sangon Biotech, Shanghai, China) to ensure that the desired mutations were correct. The primers used in this study are shown in Table S1, and information of all mutants is listed in Table S2.

### 2.2. Transformation and Inoculation of Agrobacterium

All the aforementioned mutants and the three-stranded wild-type CMV_Fny_ were transformed into the competent cells of Agrobacterium tumefaciens strain GV3101 using the freeze-thaw method [49]. Positive clones were screened via colony PCR and cultured in LB medium (50 µg/mL of kanamycin and 100 µg/mL of rifampicin) at 28 °C with shaking until the OD_600_ increased to 1.0–2.0. The bacterial solution was centrifuged at 6000× *g* 25 °C and resuspended in buffer (10 mM MgCl_2_, 10 mM 2-(N-morpholine) ethanesulfonic acid, and 0.15 mM acetosyringone) to adjust OD_600_ equal to 1.2. Agrobacterium resuspension containing RNA1 (wt), RNA2 (wt/mutant), and RNA3 (wt) were evenly mixed in equal proportions and incubated at 28 °C for 3 h. Subsequently, the mixed Agrobacterium resuspension was infiltrated into the 5th and 6th leaves of *N. benthamiana* at the 6–8 leaf stage. Each *N. benthamiana* should be injected with 1 mL. Plants inoculated with wild-type CMV_Fny_ served as positive control and with empty vector pCB301-Rz served as the mock control. *N. benthamiana* was grown at 25 °C under a 16 h light/8 h dark cycle. Each treatment includes 15 plants and is replicated three times.

### 2.3. Virus Friction Inoculation

The virus friction inoculation method was performed as follows: *N. benthamiana* leaves infected with target virus were ground into a fine homogenate using a mortar and pestle with 0.01 M phosphate-buffered saline (PBS, 137 mM NaCl, 2.68 mM KCl, 1.76 mM KH_2_PO_4_, 10 mM Na_2_HPO_4_, pH 7.2) at a ratio of 1:5 (*w*/*v*). The homogenate was then gently rubbed onto the Silicon carbide (Aladdin, Shanghai, China, S104652-1kg, ≥99%)-dusted leaves of 6–8 leaf *N. benthamiana* plants. After inoculation, the leaves were rinsed with sterile water to remove the excess corundum and homogenate on the surface of the inoculated leaves.

### 2.4. Analysis of Pathogenicity of Mutants

To assess the pathogenicity of the mutants in this study, *N. benthamiana* were observed for symptom after 14 days post-inoculation (dpi). The symptoms of plants including mosaic, leaf curling, stunting, necrosis, and chlorosis, and so on. Calculate the disease index based on the symptom standards [29,47].

Level 0: Whole plant is free of disease.

Level 1: Heart veins are bright or slightly mosaic, with no obvious stunt of the diseased plant.

Level 3: One-third of the leaves are mosaic but not malformed, or the diseased plant is stunted to more than three-fourths of the normal plant height.

Level 5: One- to two-thirds of the leaves are mosaic, or have few malformation or necrosis of the main lateral veins, or the plant is stunted to two-thirds to three-quarters of normal.

Level 7: One-half to two-thirds of the leaf leaves are mosaic, or have malformation or necrosis of the main lateral veins, or the plant is stunted to one-half to two-thirds of normal.

Level 9: All leaves are mosaic, severely malformed, or necrotic, or the plant is stunted to one-half or more of its normal height.

$$\mathrm{Disease}\;\mathrm{index}=100\;\times \;\frac{\sum (Number\;of\;diseased\;plants\;at\;each\;level\times Disease\;level)}{Total\;number\;of\;plants\times Highest\;disease\;level}$$

### 2.5. Cross-Protection Analysis

*N. benthamiana* were pre-inoculated with an attenuated mutant by Agrobacterium injection. Seven days later, the wild-type viruses were inoculated either by Agrobacterium injection or mechanical friction. At 14 days after challenge inoculation, the disease severity of tobacco plants was evaluated according to the national standard of GB/T 23222-2008 [50]. The relative protection efficiency are as follow:

$$\mathrm{Relative}\;\mathrm{control}\;\mathrm{efficacy}\;(\%)\;=\;100\%\times (1-\frac{Disease\;index\;of\;treatment\;group}{Disease\;index\;of\;control\;group})$$

### 2.6. Total RNA Extraction and PCR Detection

Select upper non-inoculated leaves sample with an approximate area of 1 cm^2^ and transfer it into a 2-mL centrifuge tube. Add sterilized steel beads and 1 mL of TransZol reagent (TransGen Biotech, Beijing, China, ER501-01-V2), then homogenize the sample using a tissue grinder. Vibrate at 70 Hz for 90 s, followed by incubation on the ice for 5 min. Add 0.2 mL of chloroform, vortex at 70 Hz for 15 s, and allow phase separation by incubating on the ice for 3 min. Centrifuge at 4 °C and 13,000× *g* for 15 min. Carefully transfer the clear upper aqueous phase to a new 1.5-mL centrifuge tube. Add 0.5 mL of isopropanol, gently invert the tube to mix, and incubate on the ice for 10 min. Centrifuge again at 4 °C and 13,000× *g* for 10 min. Discard the supernatant, leaving a gel-like RNA pellet adhered to the side and bottom of the tube. Wash the pellet with 1 mL of 75% (*v*/*v*) ethanol, vortex vigorously, and centrifuge at 4 °C and 13,000× *g* for 5 min. Carefully remove the ethanol supernatant and air-dry the pellet on a superclean bench for 5–10 min. Finally, dissolve the RNA pellet in 30–50 µL of DEPC-treated, nuclease-free water and store at −80 °C for long-term preservation.

Reverse transcription PCR was used to analysis the stability of mutants, quantitative real-time PCR was used to analysis viral accumulation. Reverse transcription was performed with M-MLV reverse transcriptase (Accurate Biology, AG11605) using random primers. PCR amplification was performed with specific primers targeting the CMV RNA2, and the amplicons were sequenced to check the mutation sites to analyze the stability of mutants. Quantitative real-time PCR (qRT-PCR) was performed using Hieff^®^ qPCR SYBR Green Master Mix (Yeasen Biotechnology, 11201ES08), with relative quantities calculated by the 2^−ΔΔCt^ method. The Actin (JQ256516.1) gene of *N. benthamiana* was used as the internal reference. The qPCR experiment was repeated 3 times with three biological replicates. Primer sequences are listed in the Table S1.

### 2.7. Western Blot

At 14 days post-inoculation, collect the uninoculated systemic leaves and grind them with liquid nitrogen. Then resuspend the ground leaf tissues in ddH_2_O, β-mercaptoethanol, and 2× SDS protein loading buffer (100 mM Tris-HCl (pH 6.8), 20% glycerol, 4% SDS, and 0.2% bromophenol blue) with a ratio of 10:1:9. The samples were denatured in a boiling water bath for 10 min, cooled on ice, and centrifuged to collect the supernatant. The extracted total protein was concentrated in a 5% SDS-PAGE gel, separated in a 12.5% SDS-PAGE gel, and then transferred onto a nitrocellulose membrane (Pall Corporation, Beijing, China). Before Western Blot, Ponceau S staining was used to quantify the proteins. Western blot analysis was performed with CMV CP antibody at a ratio of 1:5000, HRP-labeled Rabbit anti-Goat (ComWin Biotech) at a ratio of 1:5000, the target protein bands were visualized using an enhanced chemiluminescence (ECL) detection kit (NCM Biotech, P10100) and ChampChemi (SAGECREATION).

### 2.8. Statistical Analysis

The western blot signals were relatively quantified using Image J software. The data statistics were reported as the mean ± standard deviation. The data were compared and analyzed using the Mann-Whitney U test in SPSS 22.0 and “*” indicates significant difference (*p* < 0.05) between the treatment group and the control group. All data were obtained after repeating a given experiment 3 times.

## 3. Results

### 3.1. Construction of Attenuated Vaccine Vector Based on CMV RNA2

**Figure 1:**
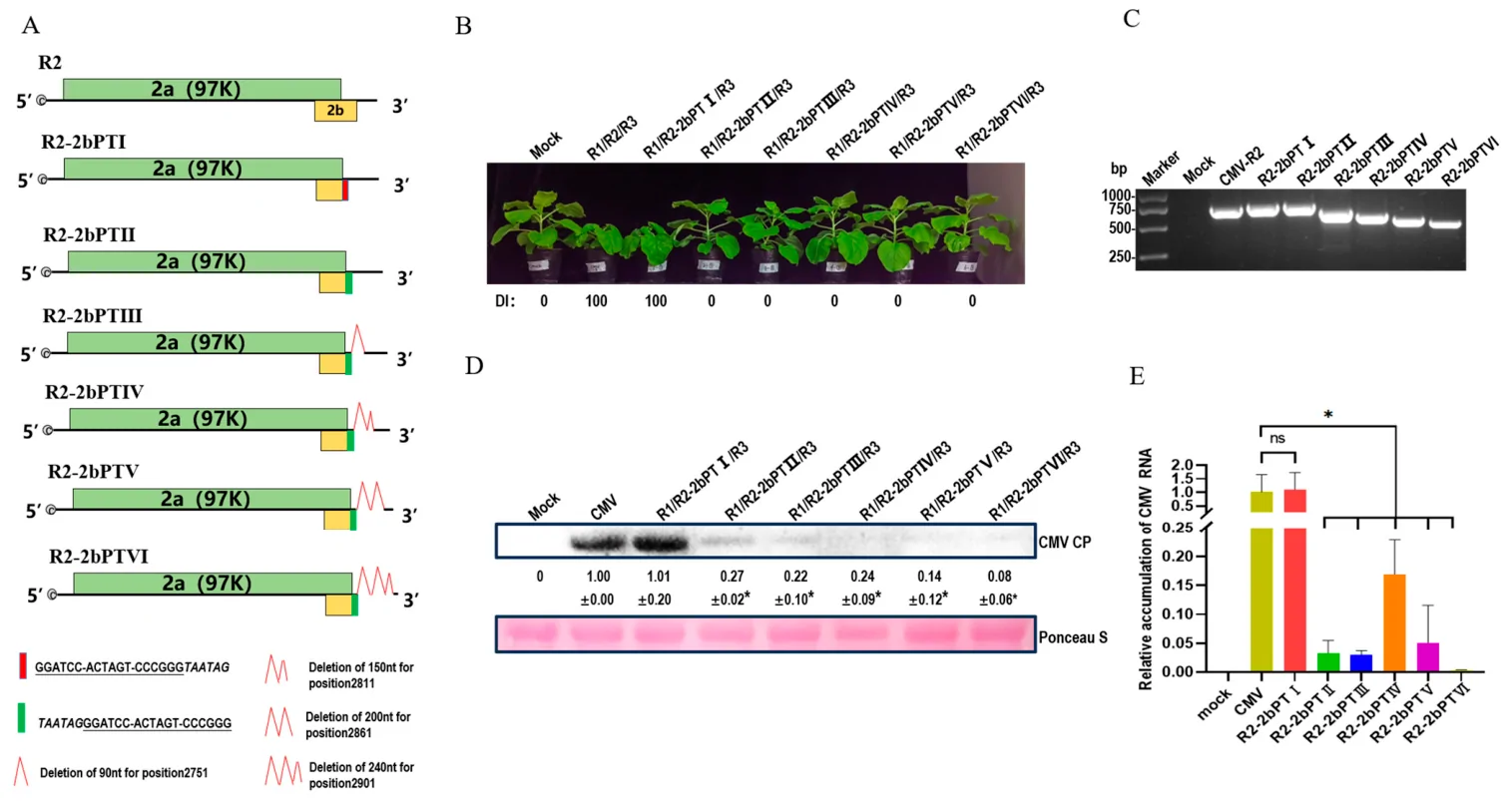
Construction and virulence analysis of CMV RNA2 mutants. (**A**) Schematic diagram of mutant of CMV RNA2. Underlined: enzyme sites, italic: stop codon. (**B**) Pathogenicity analysis of *N. benthamiana* inoculated with different CMV RNA2 mutants. DI: disease index. Mock: plant treated with Agrobacterium lacking plasmid. (**C**) Stability analysis of the mutant by RT-PCR. (**D**) Relative protein expression levels of CP by western blotting. Ponceau S: Ponceau S staining. “*” indicates significantly (*p* < 0.05). (**E**) Relative viral accumulation levels by qRT-PCR. “ns” indicates not significant. “*” indicates significantly (*p* < 0.05).

Six mutants named R2-2bPTI, R2-2bPTII, R2-2bPTIII, R2-2bPTIV, R2-2bPTV, and R2-2bPTVI were constructed by modifying the 2b coding region and 3′ UTR of CMV RNA2. The strategy is illustrated in Figure 1A.

The pathogenicity of the mutants was analyzed via Agrobacterium-mediated inoculation into *N. benthamiana*, and symptoms were observed and recorded after 14 days. Symptoms of the plants inoculated with R2-2bPTI including dwarfing, leaf wrinkling, and mosaic, similar to plants infected with CMV_Fny_ (Figure 1B)_._ The disease index (DI) was 100 and indicated that the mutant R2-2bPTI was high virulence. In contrast, the disease indexes of the plants inoculated with R2-2bPTII, R2-2bPTIII, R2-2bPTIV, R2-2bPTV, and R2-2bPTVI were 0, with no significant dwarfing and the leaves remaining flat. These symptoms were similar to negative control group, indicating weak virulence. At 14 days post-inoculation on *N. benthamiana*, total RNA was extracted, and RT-PCR was performed to evaluate the stability of each mutant. The fragment amplified from plants inoculated with CMV_Fny_ was 726 bp, while the corresponding fragment for plants inoculated with R2-2bPTI, R2-2bPTII, R2-2bPTIII, R2-2bPTIV, R2-2bPTV, and R2-2bPTVI were 750 bp, 750 bp, 660 bp, 600 bp, 550 bp, and 510 bp, respectively (Figure 1C). These results indicated that these mutants were stable.

To further assess the pathogenicity of the mutants, Western blot and qRT-PCR were performed to detect CP expression and viral accumulation in systemic leaves of plants at 14 days post-inoculation. The Western blot showed that CP expression in plants inoculated with R2-2bPTI was similar to that of the plants inoculated with CMV_Fny_. In contrast, CP expression in plants inoculated with R2-2bPTII, R2-2bPTIII, R2-2bPTIV, R2-2bPTV, and R2-2bPTVI were 0.27, 0.22, 0.24, 0.14, and 0.08 times that of CMV_Fny,_ respectively (Figure 1D). qRT-PCR showed that the viral accumulation in plants inoculated with R2-2bPTI was higher than that in plants inoculated with CMV_Fny_, being about 1.092 times that of CMV_Fny_. However, viral accumulation in plants inoculated with mutants R2-2bPTII, R2-2bPTIII, R2-2bPTIV, R2-2bPTV, and R2-2bPTVI was significantly lower than that in plants inoculated with CMV_Fny_, measuring 0.033, 0.029, 0.169, 0.051, and 0.004 fold that of CMV_Fny_ respectively (Figure 1E). These results confirmed that R2-2bPTII, R2-2bPTIII, R2-2bPTIV, R2-2bPTV, and R2-2bPTVI were stable and weak virulence with lower accumulation of both viral proteins and viral RNA.

All the results indicate that R2-2bPTII, R2-2bPTIII, R2-2bPTIV, R2-2bPTV, and R2-2bPTVI with great potential as vaccine vectors.

### 3.2. Cross-Protection of Attenuated CMV Mutants

Seven days after pre-inoculation with five attenuated mutants, *N. benthamiana* plants were challenged with wild type CMV_Fny_. At 14 days after challenged, symptoms, disease index, relative control efficacy, CMV CP expression, and CMV RNA accumulation were assessed to evaluate the cross-protection of the five attenuated mutants.

Symptoms of the *N. benthamiana* not pre-inoculated with attenuated mutant were similar to the mock control plants, showed significant dwarfing, heart leaf wrinkling, narrowed and twisted leaves with uneven coloration and dark green lesions, and brown necrosis in lower leaves. The disease index of plants inoculated with wild type CMV_Fny_(R1/R2/R3) was 100 and that of the mock control plants was 77.8.

**Figure 2:**
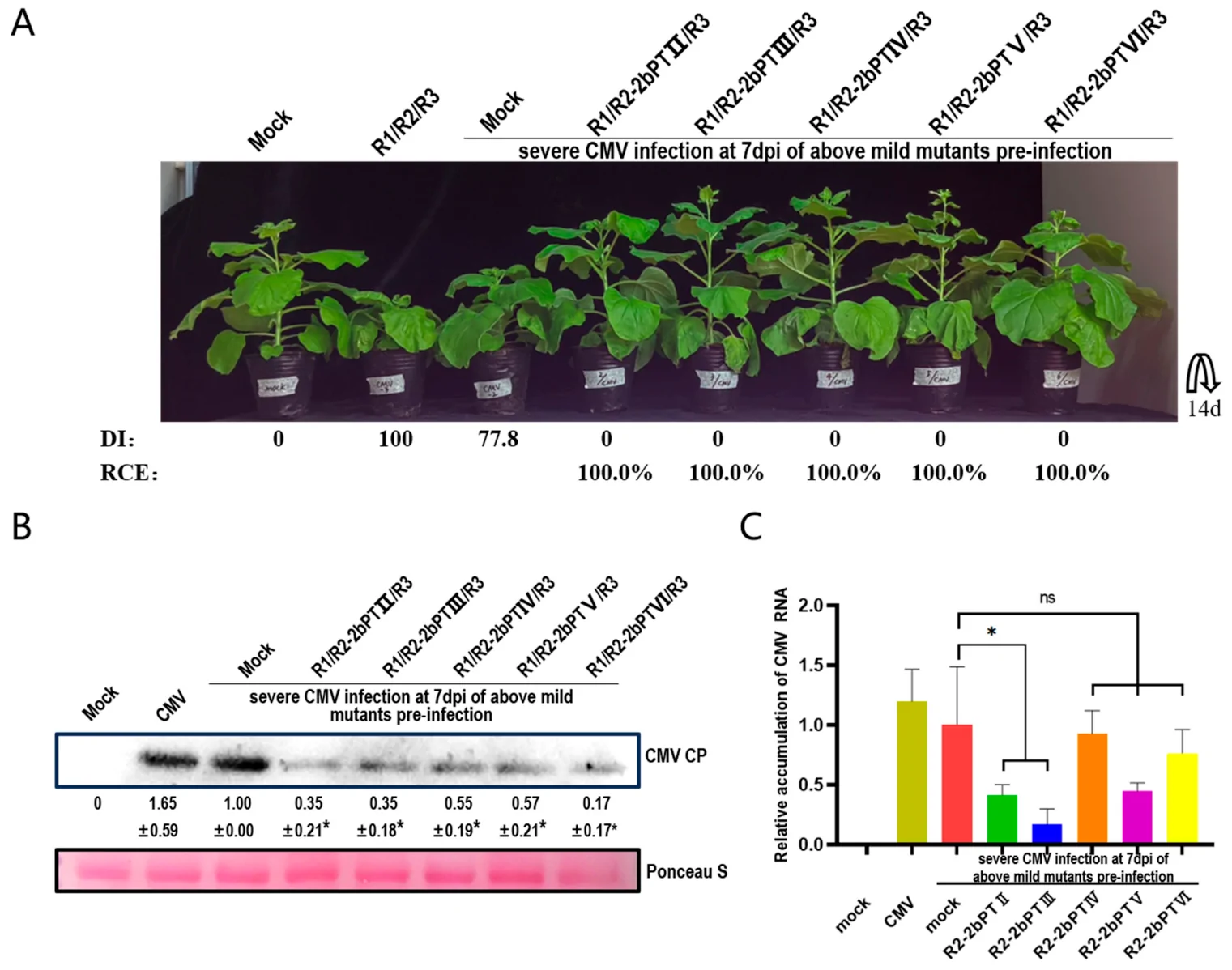
Analysis of the cross-protection effect of *N. benthamiana* pre-inoculated with attenuated mutants. (**A**) Cross-protection analysis of *N. benthamiana* inoculated with different CMV RNA2 mutants. DI: disease index. REC: Relative control efficacy. Mock: plant treated with Agrobacterium lacking plasmid. dpi: days post inoculation. (**B**) Relative protein expression levels of CP by western blotting. Ponceau S: Ponceau S staining. “*” indicates significantly (*p* < 0.05). (**C**) Relative viral accumulation levels by qRT-PCR. “ns” indicates not significant. “*” indicates significantly (*p* < 0.05).

In contrast, plants inoculated with the attenuated mutant maintained normal height with horizontally spread leaves, similar to the mock control plants. All of them showed a disease index of 0 and a relative control efficacy of 100% (Figure 2A). Western blot analysis of the CMV CP expression revealed that plants pre-inoculated with attenuated mutants showed significantly lower than non-pre-inoculated plants. The expression of CMV CP in plants pre-inoculated with the attenuated mutants R2-2bPTII, R2-2bPTIII, R2-2bPTIV, R2-2bPTV, and R2-2bPTVI were 0.35, 0.35, 0.55, 0.57, and 0.17 fold that of mock plants control non-pre-inoculated with attenuated mutants, respectively (Figure 2B). qRT-PCR analysis of CMV RNA accumulation revealed that plants pre-inoculated with attenuated mutants showed significantly lower than non-pre-inoculated plants. The accumulation of CMV RNA in plants pre-inoculated with attenuated mutants R2-2bPTII, R2-2bPTIII, R2-2bPTIV, R2-2bPTV, and R2-2bPTVI was 0.42, 0.17, 0.93, 0.45, and 0.76 fold that of mock plants control non-pre-inoculated with attenuated mutants, respectively (Figure 2C). These findings suggest that pre-inoculation with the attenuated mutants R2-2bPTII, R2-2bPTIII, R2-2bPTIV, R2-2bPTV, and R2-2bPTVI can effectively attenuate disease symptoms and significantly suppress the accumulation of both viral proteins and viral RNA.

### 3.3. Capacity of RNA2 Mutants to Accommodate Exogenous Fragments of Different Lengths

**Figure 3:**
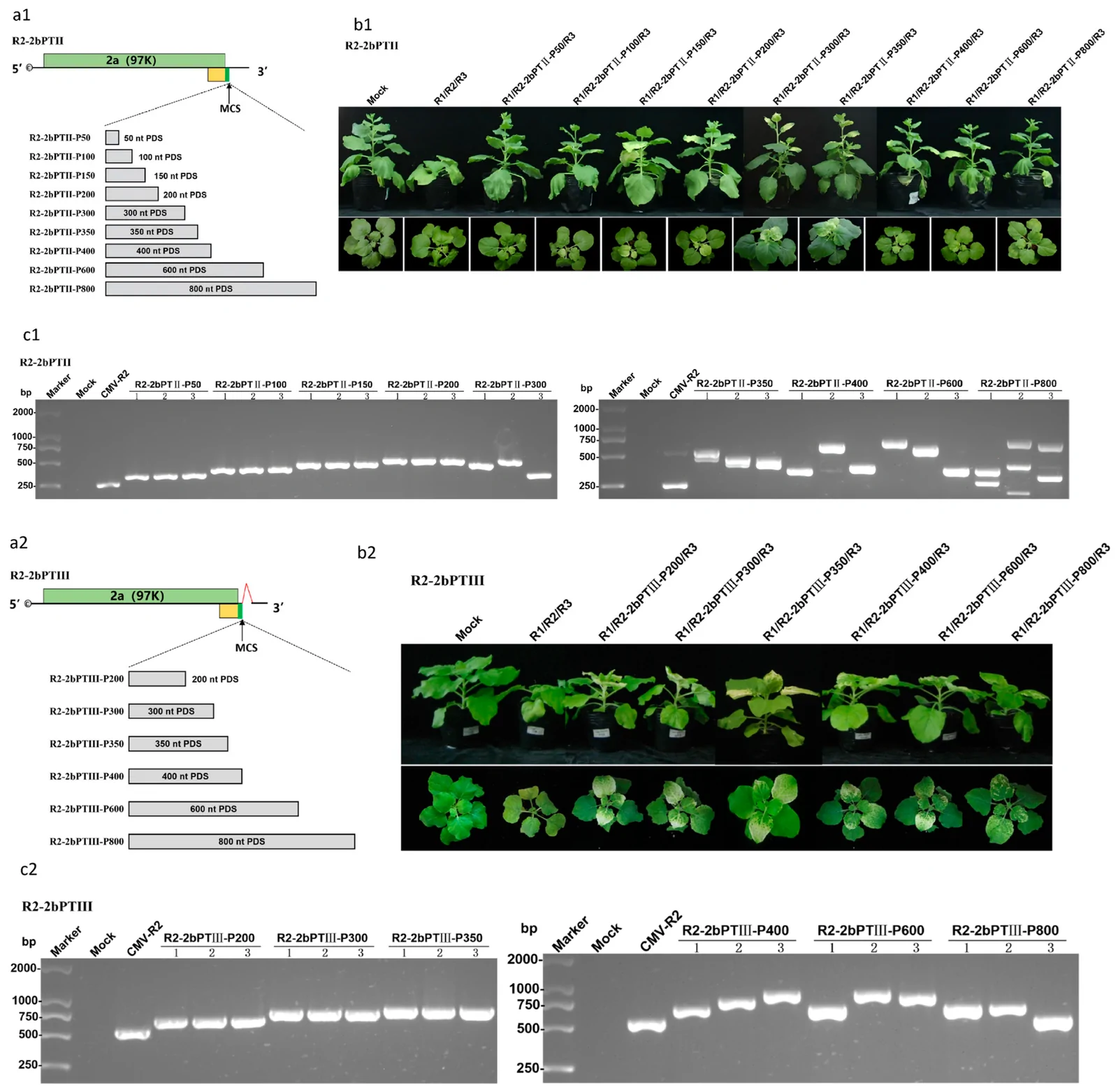

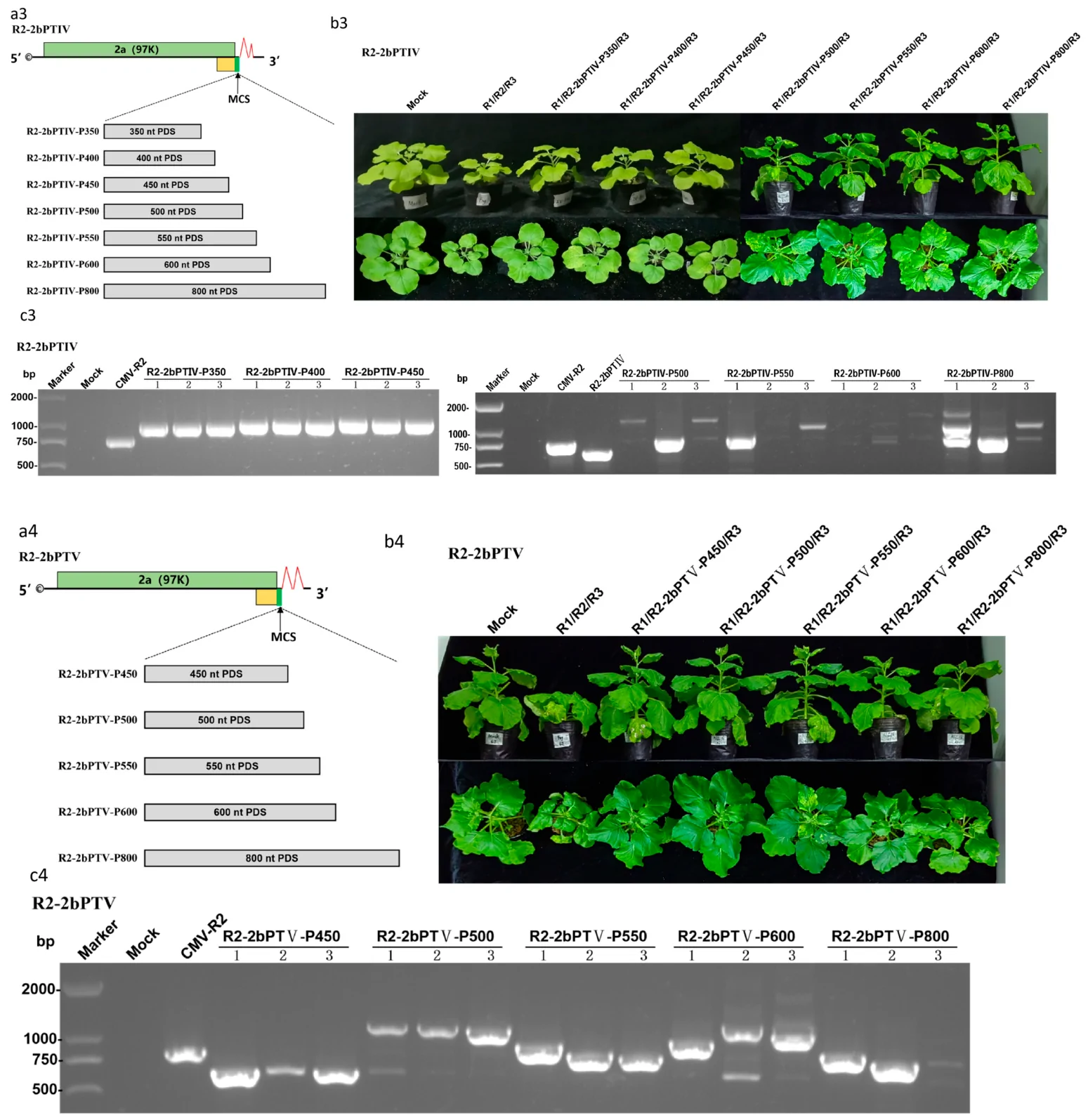

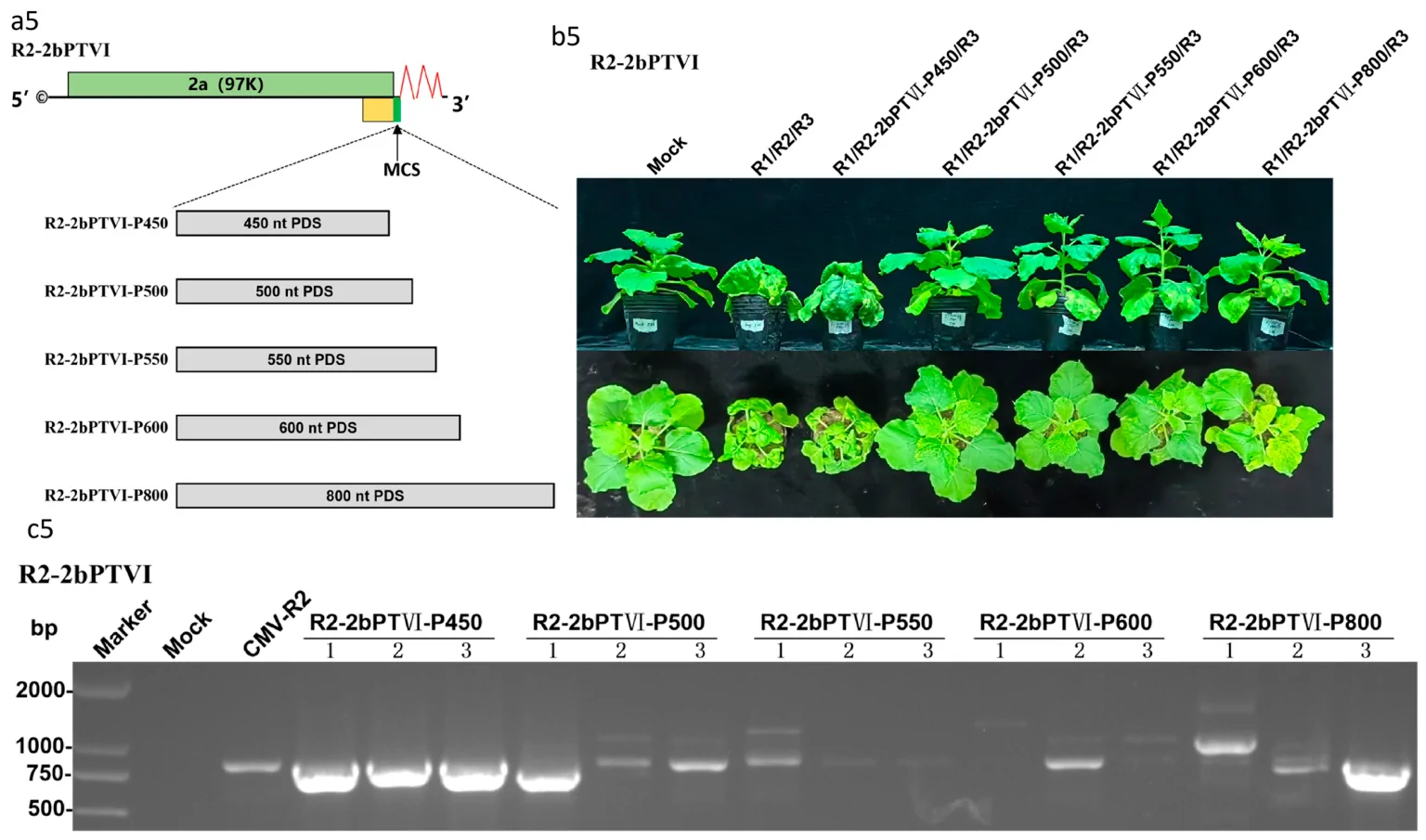
Analysis of the ability of RNA2 mutants to accommodate exogenous fragments of different lengths. (**a1**–**a5**) Schematic diagram of CMV RNA2 mutants with PDS inserts of different lengths. (**b1**–**b5**) Analysis of silencing phenotypes in CMV RNA2 mutants with PDS inserts of different lengths. (**c1**–**c5**) RT-PCR analysis of the stability of mutants carrying PDS fragments.

Phytoene desaturase (PDS) is a key enzyme in the carotenoid biosynthesis pathway, and silencing of the *PDS* gene results in a leaf-bleaching silencing phenotype in plants. Therefore, the success of gene silencing can be verified by the occurrence of leaf bleaching. In this study, the *PDS* gene fragment was chosen as a reporter to assess the ability of RNA2 mutants to accommodate exogenous fragments. *PDS* gene fragments of different lengths were individually inserted into the multiple cloning site (MCS) of the attenuated CMV RNA2 mutant vector. The recombinant mutants carrying PDS fragments of different lengths were then inoculated into *N. benthamiana* leaves via agroinfiltration. At 14 days, bleaching symptoms in systemic leaves were investigated to assess the capacity of RNA2 mutants to accommodate exogenous fragments. A schematic diagram of the mutant construction strategy is shown in Figure 3(a1–a5). The statistical analysis of systemic leaf bleaching phenotypes showed that different-length PDS fragment insertion mutants cause varying degrees of leaf bleaching (Figure 3(b1–b5)), and the degree of leaf bleaching is not related to the length of *PDS*. Importantly, *N. benthamiana* leaves inoculated with R2-2bPTII-P50 showed only slight bleaching, while those inoculated with R2-2bPTII-P100 exhibited a significant bleaching, suggesting that the minimum insert length of exogenous fragments required to produce a silencing phenotype is 100 bp (Figure 3(b1)).

To further access the stability of the mutants carrying PDS fragments, RT-PCR was performed to detect the integrity of the inserted PDS gene fragments in different mutants (Figure 3(c1–c5)). The amplification fragment of the wild-type is approximately 250 bp. If R2-2bPTII-P50, R2-2bPTII-P100, R2-2bPTII-P150, R2-2bPTII-P200, R2-2bPTII-P300, R2-2bPTII-P350, R2-2bPTII-P400, R2-2bPTII-P600, and R2-2bPTII-P800 are stably, the amplification fragments will be approximately 300 bp, 350 bp, 400 bp, 450 bp, 550 bp, 600 bp, 650 bp, 850 bp, and 1050 bp respectively (Figure 3(c1)). The amplification fragment of the wild-type is approximately 540 bp. If R2-2bPTIII-P200, R2-2bPTIII-P300, R2-2bPTIII-P350, R2-2bPTIII-P400, R2-2bPTIII-P600, and R2-2bPTIII-P800 are stably, the amplification fragments will be approximately 670 bp, 770 bp, 820 bp, 870 bp, 1070 bp, and 1270 bp respectively (Figure 3(c2)). The amplification fragment of the wild-type is approximately 726 bp. If R2-2bPTIV-P350, R2-2bPTIV-P400, R2-2bPTIV-P450, R2-2bPTIV-P500, R2-2bPTIV-P550, R2-2bPTIV-P600, and R2-2bPTIV-P800 are stably, the amplification fragments will be approximately 944 bp, 994 bp, 1044 bp, 1094 bp, 1144 bp, 1194 bp, and 1394 bp respectively (Figure 3(c3)). The amplification fragment of the wild-type is approximately 776 bp. If R2-2bPTV-P450, R2-2bPTV-P500, R2-2bPTV-P550, R2-2bPTV-P600, and R2-2bPTV-P800 are stably, the amplification fragments will be approximately 1044 bp, 1094 bp, 1144 bp, 1194 bp, and 1394 bp respectively (Figure 3(c4)). The amplification fragment of the wild-type is approximately 816 bp. If R2-2bPTVI-P450, R2-2bPTVI-P500, R2-2bPTVI-P550, R2-2bPTVI-P600, and R2-2bPTVI-P800 are stably, the amplification fragments will be approximately 1044 bp, 1094 bp, 1144 bp, 1194 bp, and 1394 bp respectively (Figure 3(c5)). If the mutant is unstable, there will be an or more amplification fragments that are smaller than expected (Figure 3c). Therefore the upper length limits in different attenuated mutants were 200 bp for R2-2bPTII, 350 bp for R2-2bPTIII, 450 bp for R2-2bPTIV, less than 450 bp for R2-2bPTV, and less than 450 bp for R2-2bPTVI (Figure 3c). The results showed that the stability of different attenuated mutants in accommodating PDS fragments of varying lengths varied. Combined with the leaf bleaching data, the upper limit of exogenous fragments that could be stably accommodated and the potential for targeting viral species other than CMV by different attenuated mutants were clarified.

The characteristics of CMV RNA2 Mutants, including pathogenicity, stability, cross-protection, the maximum insert length of exogenous fragments, species for targeting viral beyond CMV, were listed in Table 1.

### 3.4. Application of Attenuated Mutant R2-2bPTIV

**Figure 4:**
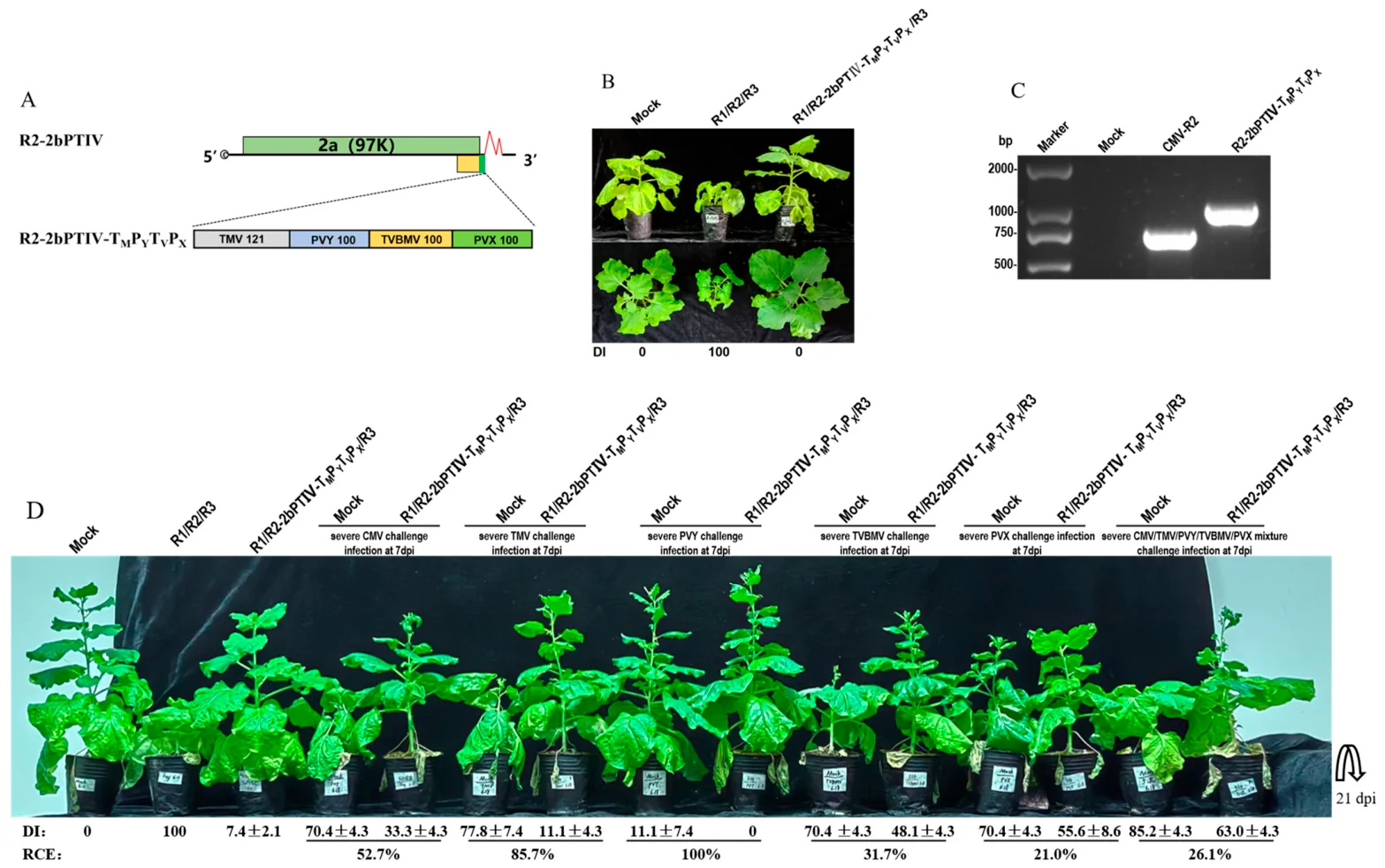
Analysis of the application potential of attenuated mutant R2-2bPTIV. (**A**) Schematic diagram of R2-2bPTIV-T_M_P_Y_T_V_P_X_. (**B**) Pathogenicity analysis of *N. benthamiana* inoculated with R2-2bPTIV-T_M_P_Y_T_V_P_X_. DI: disease index. Mock: plant treated with Agrobacterium lacking plasmid. (**C**) Stability analysis of R2-2bPTIV-T_M_P_Y_T_V_P_X_ by RT-PCR. (**D**) Cross-protection analysis of *N. benthamiana* inoculated with R2-2bPTIV-T_M_P_Y_T_V_P_X_. DI: disease index. REC: Relative control efficacy. Mock: plant treated with Agrobacterium lacking plasmid. dpi: days post inoculation.

To further verify that the attenuated mutant constructed in this study could serve as a foundational vector for attenuated vaccine development, a mutant, R2-2bPTIV-T_M_P_Y_T_V_P_X_, was constructed based on the attenuated mutant R2-2bPTIV (Figure 4A). R2-2bPTIV-T_M_P_Y_T_V_P_X_ contains fragments of TMV, PVY, TVBMV, and PVX, and can silence the target viruses CMV, TMV, PVY, TVBMV, and PVX.

Symptoms were observed 14 days after agroinfiltration into *N.* *benthamiana* leaves. Plants inoculated with CMV_Fny_ exhibited severe stunting and leaf curling and wrinkling, whereas plants inoculated with R2-2bPTIV-T_M_P_Y_T_V_P_X_ showed no obvious symptoms and appeared similar to mock-inoculated plants (Figure 4B). This indicates that R2-2bPTIV-T_M_P_Y_T_V_P_X_ is an attenuated mutant.

Stability testing of systemic leaves was performed using RT-PCR. RT-PCR amplification showed that the fragment from the systemic leaves of plants inoculated with R2-2bPTIV-T_M_P_Y_T_V_P_X_ was approximately 1014 bp. In contrast, the fragment from the CMV_Fny_-inoculated plants was approximately 726 bp (Figure 4C).

To evaluate the cross-protection of R2-2bPTIV-T_M_P_Y_T_V_P_X_ against CMV, TMV, PVY, TVBMV, and PVX, *N. benthamiana* plants pre-inoculated with R2-2bPTIV-T_M_P_Y_T_V_P_X_ were challenge-inoculated with the corresponding virulent viruses separately and in mixtures at 7 days post-initial inoculation. The cross-protection effect was analyzed 21 days later (Figure 4D).

The *N. benthamiana* plants directly inoculated with CMV_Fny_ without prior inoculation with R2-2bPTIV-T_M_P_Y_T_V_P_X_ showed severe stunting and leaf curling and wrinkling, with a disease index of 70.4. In contrast, the plants pre-inoculated with R2-2bPTIV-T_M_P_Y_T_V_P_X_ grew healthily and similar to the mock-inoculated plants. The disease index decreased to 33.3, and the relative efficacy against CMV reached 52.7%.

Similarly, *N. benthamiana* plants pre-inoculated with R2-2bPTIV-T_M_P_Y_T_V_P_X_ challenged with TMV alone, PVY alone, TVBMV alone, and PVX alone all showed mild stunting and leaf flattening. The disease indexes decreased from 77.8 to 11.1, 11.1 to 0, 70.4 to 48.1, and 70.4 to 55.6, respectively. The relative efficacy against TMV, PVY, TVBMV, and PVX reached 85.7%, 100%, 31.7%, and 21.0%, respectively.

*N. benthamiana* plants without prior inoculation with R2-2bPTIV-T_M_P_Y_T_V_P_X_ inoculated with CMV_Fny_, TMV, PVY, TVBMV, and PVX in mixtures showed severe stunting and leaf curling and wrinkling, with a disease index of 85.2. In contrast, plants pre-inoculated with R2-2bPTIV-T_M_P_Y_T_V_P_X_ grew healthily, showing mild stunting and leaf flattening. The disease index decreased to 63.0, and the relative efficacy reached 26.1%.

In conclusion, the attenuated R2-2bPTIV-T_M_P_Y_T_V_P_X_ exhibited short-term stability and partial cross-protection against target viruses. These findings confirm that the attenuated mutant R2-2bPTIV can serve as a foundational vector for attenuated vaccine development.

## 4. Discussion

### 4.1. Cross-Protection for Attenuated Vaccine

Cross-protection refers to the phenomenon that plants which infected by an attenuated strain of a virus, could resistant to subsequent infection by virulent strains of the same or closely related viruses [15,16]. This phenomenon has been widely recognized and applied in plant viral disease control as exemplified by the control of citrus tristeza virus (CTV) [20,21], Pepino mosaic virus (PepMV) [22] and zucchini yellow mosaic virus (ZYMV) [24].

The precise mechanism underlying cross-protection has not yet been clearly clarified, and referred as a complex, multifaceted process. There are several opinions on the mechanism of cross-protection such as the coat-protein mediated mechanism, RNA silencing mechanisms (RNAi), superinfection exclusion (SIE) and so on [51,52,53]. Among them, RNAi is the most widely accepted core mechanism. RNA silencing serves as the core mechanism responsible for the specificity of cross-protection in most systems, whereas coat-protein mediated and SIE as complementary or alternative mechanisms that dominate cross-protection in specific virus–host interaction contexts [54]. In this study, the cross-protective effects of the RNA fragment-inserted mutant R2-2bPTIV-T_M_P_Y_T_V_P_X_ against the virulent challenges of TMV, PVY, TVBMV, and PVX were attributed to the inserted 100 bp conserved sequence. The mechanism is predominantly associated with RNA silencing. However, this study lacks analysis of siRNA abundance and the activity of key components involved in the RNA interference pathway to certify this mechanism.

In addition, plant microbial pathogens impair plant growth and development by releasing three categories of chemical effectors, namely extracellular enzymes, secondary metabolites, and phytohormones, which play critical roles in facilitating host plant infection [55]. Whether these effector molecules secreted by the tested pathogenic viruses interfere with the cross-protective effect of R2-2bPTIV-T_M_P_Y_T_V_P_X_, and whether the attenuated vaccine can maintain stable resistance under complex pathogen co-infection conditions in the field also remains to be further explored in subsequent experiments.

### 4.2. Vectors of Attenuated Vaccine

The traditional development of attenuated vaccines relied heavily on natural mutations, which is a time-consuming process and with extensive labor for identification and isolation [56]. In addition, the attenuated vaccines obtained usually exhibited a narrow application scope, and often only providing protection against a single viral strain or a closely related subgroup [57]. Although some modern platforms of viral vector systems are widely used and can carry heterologous pathogen-derived fragments to induce broad-spectrum resistance against multiple viruses, most of these viral vectors possess low RNA silencing efficiency and poor stability. For example, the system based on potato virus X (PVX) exhibits low RNA silencing efficiency and a short silencing duration [58]. Furthermore, the tobacco rattle virus (TRV)-based system shows evident limitations in host adaptability and tissue silencing capacity [59].

In contrast, the attenuated vaccine developed in this study through precise gene editing. This approach significantly shortens the development cycle compared to traditional methods. More importantly, it exhibits exceptional target specificity, enabling the precise silencing of target gene fragments to control target viruses. An excellent vector is critical for attenuated vaccine development. In this study, CMV was selected as the vaccine vector to edit for its broad host range and multipartite genome [33,34,35]. These characteristics of CMV make it a naturally advantageous vector for satisfying the broad-spectrum and multi-target requirements of attenuated vaccine. We aimed at the CMV_Fny_ RNA2 especially the 2b protein reading frame, which is a suppressor of viral gene silencing that is closely related to viral pathogenicity in plants [39,40]. By precisely modifying the 2b protein reading frame, we successfully attenuated the viral pathogenicity while retaining its ability to systemically infect the host and express the inserted gene silencing fragments. Subsequent work could involve engineering RNA1 and RNA3 of CMV_Fny_ to develop an attenuated vaccine vector with greater carrying capacity and cross-protection efficacy.

### 4.3. Exogenous Insert of the Attenuated Vaccine

The maximum exogenous fragment capacity represents a key performance indicator for viral vectors. The insertion length of exogenous fragments is not determined merely by fragment length but is also affected by fragment sequence characteristics and structures formed after insertion.

In this study we found that the cross-protection effect is affected significantly by both the arrangement order and the length of inserted fragments. For plant viruses, gene sequence rearrangement significantly modulates viral gene expression, symptom severity, and host adaptability. For example, rearrangements of the CP gene and MP gene in TMV could alter its systemic movement efficiency and pathogenicity significantly in *N. benthamiana* [60].

Two RNA2 mutants R2-2bPTI and R2-2bPTII constructed in this study, which differ only in the inserted termination codon sequences and multiple cloning site (MCS) in the end of 2b protein. Nevertheless, this difference directly leads to significant disparities in pathogenicity, viral CP protein translation efficiency, and viral RNA accumulation between the two mutants. The R2-2bPTI exhibits no difference from CMV_Fny_, whereas R2-2bPTII showed markedly attenuated virulence, making it suitable as a vector for attenuated vaccines. This finding highlights the flexibility and adaptability of our RNAi-based strategy for developing attenuated vaccines. And also suggests that the vaccine efficacy can be optimized via gene sequence rearrangement. In the future, we will pay attention to field applications, making a safe, convenient, stable and reliable commercial product.

This enables the development of a single vaccine that can target multiple viruses simultaneously, addressing the single target limitation of conventional attenuated vaccines.

### 4.4. Application of Attenuated Vaccine

Application of modified viruses for plant protection may pose biosafety risks. Genetic recombination and heterologous packaging constitute important evolutionary mechanisms that generate viral diversity, as well as core biosafety risks [61,62].

In this study, CMV was selected as the attenuated vaccine vector because of its diverse host range and distinct symptomatic manifestations, which facilitates the evaluation of research results and exploration of application potential. However, the CMV genome consists of three +ssRNA segments, and this multipartite nature renders CMV more prone to genetic recombination between its different genes and distinct viral strains. Before field-scale deployment, multi-generational inoculation trials should be conducted to continuously monitor the attenuated vaccine’s genomic stability, determine the probability of recombination and the potential spectrum of genetic variation, and assess its potential impact on the environment and crop performance.

At present, the attenuated vaccine constructed in this research has only been tested for the disease resistance and short-term safety and stability and in *N. benthamiana*. Its efficacy in real hosts has not been established and cannot be directly extrapolated from data generated using this model plant. Multi-regional field trials covering diverse cropping seasons are needed to determine its protective performance and applicable scenarios under varied climatic conditions and for different major crop varieties, and to identify suitable application methods and timing.

## 5. Conclusions

In this study, five potential RNA-virus-targeted attenuated vaccine vectors were constructed via gene editing strategies based on gene silencing. Simultaneously, the permissible insert fragment size range for each vector to achieve efficient gene silencing was evaluated using PDS constructs of different lengths. A pentavalent attenuated vaccine targeting CMV, TMV, TVBMV, PVX and PVY was developed based on the attenuated vector R2-2bPTIV. This vaccine showed partial protective efficacy against the target viruses and displayed favorable short-term safety and stability in *N. benthamiana*. This study provides materials for the artificial construction of plant attenuated vaccines and offers material and technical support for the development of single-dose multi-target vaccines.

## Figures and Tables

**Table 1:** Characteristics of CMV RNA2 Mutants.

Mutant	Pathogenicity	Stability	Cross-Protection	The Maximum Insert Length of Exogenous Fragments	Species for Targeting Viral Beyond CMV
R2-2bPTI	virulent	Yes	Not Applicable	/	/
R2-2bPTII	attenuated	Yes	Applicable	200 bp	2
R2-2bPTIII	attenuated	Yes	Applicable	350 bp	3
R2-2bPTIV	attenuated	Yes	Applicable	450 bp	4
R2-2bPTV	attenuated	Yes	Applicable	<450bp	N/A
R2-2bPTVI	attenuated	Yes	Applicable	<450bp	N/A

## Data Availability

The datasets used and/or analyzed during the current study available from the corresponding author on reasonable request.

## Supplementary Materials

The following supporting information can be downloaded at: https://www.mdpi.com/article/10.3390/life16091489/s1, Table S1: Primers used in this study; Table S2: Information of mutants constructed in this study.
