# Supplementary material for: Construction and Characterization of an Attenuated Vaccine Vector Based on Cucumber Mosaic Virus RNA2
Source: Life (Basel). 2026 Sep 5;16(9):1489. doi: 10.3390/life16091489 (PMC13608468; doi:10.3390/life16091489)
Supplement: Supplementary file 1 [file life-16-01489-s001.zip › Table S1.pdf]

Table S1. Primers used in this study

**Primer name**	**Sequence（5’→3’）**	**Purpose**
del-2b 3’-BSST-2662-F	*TC*<u>GGATCCACTAGTCCCGGG</u>**TAATA**GCCTCTCGTTTAGAGTTATCGGCG	Construction of R2-2bPTI
del-2b 3’-B-2661-R	*AT*<u>GGATCC</u>CTCAGACTCGGGTAACTCCGCC	Construction of R2-2bPTI
Fny2-del2b-2662-F	*TC*<u>GGATCCACTAGTCCCGGG</u>GCCTCTCGTTTAGAGTTATCGGCG	Construction of R2-2bPTII
Fny2-del2b-2661-R	*AT*<u>GGATCC</u>**CTATTA**CTCAGACTCGGGTAACTCCGCC	Construction of R2-2bPTII, R2-2bPTIII, R2-2bPTIV, R2-2bPTV and R2-2bPTVI
Fny2-del2b-BSS-2752-F	*TC*<u>GGATCCACTAGTCCCGGG</u>AACCTCCCCTTCCGCATCT	Construction of R2-2bPTIII
Fny2-del2b-BSS-2812-F	*TC*<u>GGATCCACTAGTCCCGGG</u>CTATAAACTGTCTGAAGTCACTAAACA	Construction of R2-2bPTIV
Fny2-del2b-BSS-2862-F	*TC*<u>GGATCCACTAGTCCCGGG</u>TCCAGCTTACGGCTAAAATGG	Construction of R2-2bPTV
Fny2-del2b-BSS-2902-F	*TC*<u>GGATCCACTAGTCCCGGG</u>CGCCAGCAGACTTACAAGT	Construction of R2-2bPTVI
PDS-Sma I-333-F	*AT*<u>CCCGGG</u>GGAGATTGTTATTGCTGGTGCAGG	Amplification of PDS (DQ469932) of *N. benthamiana*
PDS-BamH I-382-R	*AT*<u>GGATCC</u>ATTTTGCTGTAGACAAACCACCC	Construction of R2-2bPTII-P50
PDS-BamH I-432-R	*AT*<u>GGATCC</u>GACATCTCTTGCCTCCAGC	Construction of R2-2bPTII-P100
PDS-BamH I-482-R	*AT*<u>GGATCC</u>TCGTACCAATCTCCATCATCATCT	Construction of R2-2bPTII-P150
PDS-BamH I-531-R	*AT*<u>GGATCC</u>CAGGTTCTGCATATTTGGGTAAGC	Construction of R2-2bPTII-P200 and R2-2bPTIII-P200
PDS-BamH I-632-R	*AT*<u>GGATCC</u>GGAAAATCAAAGCGGCTGAACT	Construction of R2-2bPTII-P300 and R2-2bPTIII-P300
PDS-BamH I-682-R	*AT*<u>GGATCC</u>TGTTCTTTAGTATGGCCAAAATTCCA	Construction of R2-2bPTII-P350, R2-2bPTIII-P350 and R2-2bPTIV-P350
PDS-BamH I-732-R	*AT*<u>GGATCC</u>CAAGAGTCCAATAGCAAATTTGACT	Construction of R2-2bPTII-P400, R2-2bPTIII-P400 and R2-2bPTIV-P400
PDS-BamH I-782-R	*AT*<u>GGATCC</u>CTTAAACCGTCTTGAGCTTCAACA	Construction of R2-2bPTIV-P450, R2-2bPTV-P450 and R2-2bPTVI-P450
PDS-BamH I-832-R	*AT*<u>GGATCC</u>CATCTGTCACCCTATCAGGCA	Construction of R2-2bPTIV-P500, R2-2bPTV-P500 and R2-2bPTVI-P500
PDS-BamH I-882-R	*AT*<u>GGATCC</u>CTCGTCAGGGTTTATGAAGTTAAGTG	Construction of R2-2bPTIV-P550, R2-2bPTV-P550 and R2-2bPTVI-P550
PDS-BamH I-932-R	*AT*<u>GGATCC</u>TTCTCCTGAAGAAATCTGTTCAAAGC	Construction of R2-2bPTII-P600, R2-2bPTIII-P600, R2-2bPTIV-P600, R2-2bPTV-P600 and R2-2bPTVI-P600
PDS-BamH I-1132-R	*AT*<u>GGATCC</u>CAAACACAAAAGCATCTCCTTTAATTGT	Construction of R2-2bPTII-P800, R2-2bPTIII-P800, R2-2bPTIV-P800, R2-2bPTV-P800 and R2-2bPTVI-P800
TMV-BamH I-2158-F	*AT*<u>GGATCC</u>ACACGGGTCCGATTAAAGTTCA	Construction of R2-2bPTIV-$T_{M}P_{Y}T_{V}P_{X}$
TMV-cz-pvy-2278-R	GGCTCTCCTCAAGGTCAATAGCAGCTGTATCT	
PVY-cz-tmv-517-F	GACCTTGAGGAGAGCCACCTTCAAAACTTG	
PVY-cz-tvbmv-616-R	AGGTGCTGTCAACTTTGGCGTGTGATATGTTT	
TVBMV-cz-pvy-7600-F	AAAGTTGACAGCACCTCTGGATACTCTACTAG	
TVB-7699-CZ-PVX-R	TCCAACAGATCCCGACACTCCATGGTC	
PVX-1967-CZ-TVB-F	GTCGGGATCTGTTGGATTCAAGGCGCTAG	
PVX-2066-Sma I-R	*AT*<u>CCCGGG</u>GGGAAGTCCTCTTTTTCCAACC	

Primer name	Sequence（5'→3'）	Purpose
CMV-Fny2-2324-F	TTATTCAGATCGTCGTCAGTGC	Detection of CMV-R2 mutants
CMV-Fny2-2274-F	GGCGCCTTTAAGAAGTATACCG	
CMV-Fny2-2234-F	GAAATACAAGAAGTCTGGGGAAGA	
CMV-Fny2-2510-F	AGAATCGACGGGAACGAGGT	
CMV-Fny2-2760-R	GGGGAGGTTTCAGAAAGCACC	
CMV-Fny2-3050-R	TGGTCTCCTTTTGGAGGCC	
qNbActin-F	CAAGGAAATCACCGCTTTGG	Used for qRT-PCR
qNbActin-R	AAGGGATGCGAGGATGGA	
qCMV-CP-171-F	CTTTGTAGGGAGTGAACGCT	
qCMV-CP-369-R	CACCCACACGGTAGAATCAA	

Note: protection nuclectides: Italic; enzyme sites: underlined; stop codon: bold
