# Supplementary material for: Construction and Characterization of an Attenuated Vaccine Vector Based on Cucumber Mosaic Virus RNA2
Source: Life (Basel). 2026 Sep 5;16(9):1489. doi: 10.3390/life16091489 (PMC13608468; doi:10.3390/life16091489)
Supplement: Supplementary file 1 [file life-16-01489-s001.zip › Table S2.pdf]

Table S2.Information of mutants constructed in this study

**Name**	**Mutant site information**	**Purpose**
R2-2bPTI	Fragments inserted downstream of ^{2661}G of CMV$_{Fny}$-RNA2:GGATCCACTAGTCCCGGG**TAATAG**	
R2-2bPTII	Fragments inserted downstream of ^{2661}G of CMV$_{Fny}$-RNA2:**TAATAG**GGATCCACTAGTCCCGGG	
R2-2bPTIII	Fragments inserted downstream of ^{2661}G of CMV$_{Fny}$-RNA2:**TAATAG**GGATCCACTAGTCCCGGG and deletion of the fragment at positions 2662–2751.	
R2-2bPTIV	Fragments inserted downstream of ^{2661}G of CMV$_{Fny}$-RNA2:**TAATAG**GGATCCACTAGTCCCGGG and deletion of the fragment at positions 2662-2811.	Construction of Attenuated Mutants
R2-2bPTV	Fragments inserted downstream of ^{2661}G of CMV$_{Fny}$-RNA2:**TAATAG**GGATCCACTAGTCCCGGG and deletion of the fragment at positions 2662-2861.	
R2-2bPTVI	Fragments inserted downstream of ^{2661}G of CMV$_{Fny}$-RNA2:**TAATAG**GGATCCACTAGTCCCGGG and deletion of the fragment at positions 2662-2901.	
R2-2bPTII-P50	The 50 bp PDS fragment was inserted in reverse orientation between the BamHI and SmaI restriction sites of R2-2bPTII.	
R2-2bPTII-P100	The 100 bp PDS fragment was inserted in reverse orientation between the BamHI and SmaI restriction sites of R2-2bPTII.	
R2-2bPTII-P150	The 150 bp PDS fragment was inserted in reverse orientation between the BamHI and SmaI restriction sites of R2-2bPTII.	
R2-2bPTII-P200	The 200 bp PDS fragment was inserted in reverse orientation between the BamHI and SmaI restriction sites of R2-2bPTII.	Assessment Ability of R2-2bPTII to accommodate exogenous fragments
R2-2bPTII-P300	The 300 bp PDS fragment was inserted in reverse orientation between the BamHI and SmaI restriction sites of R2-2bPTII.	
R2-2bPTII-P350	The 350 bp PDS fragment was inserted in reverse orientation between the BamHI and SmaI restriction sites of R2-2bPTII.	
R2-2bPTII-P400	The 400 bp PDS fragment was inserted in reverse orientation between the BamHI and SmaI restriction sites of R2-2bPTII.	
R2-2bPTII-P600	The 600 bp PDS fragment was inserted in reverse orientation between the BamHI and SmaI restriction sites of R2-2bPTII.	
R2-2bPTII-P800	The 800 bp PDS fragment was inserted in reverse orientation between the BamHI and SmaI restriction sites of R2-2bPTII.	
R2-2bPTIII-P200	The 200 bp PDS fragment was inserted in reverse orientation between the BamHI and SmaI restriction sites of R2-2bPTIII.	
R2-2bPTIII-P300	The 300 bp PDS fragment was inserted in reverse orientation between the BamHI and SmaI restriction sites of R2-2bPTIII.	Assessment Ability of R2-2bPTIII to accommodate exogenous fragments
R2-2bPTIII-P350	The 350 bp PDS fragment was inserted in reverse orientation between the BamHI and SmaI restriction sites of R2-2bPTIII.	
R2-2bPTIII-P400	The 400 bp PDS fragment was inserted in reverse orientation between the BamHI and SmaI restriction sites of R2-2bPTIII.	
R2-2bPTIII-P600	The 600 bp PDS fragment was inserted in reverse orientation between the BamHI and SmaI restriction sites of R2-2bPTIII.	
R2-2bPTIII-P800	The 800 bp PDS fragment was inserted in reverse orientation between the BamHI and SmaI restriction sites of R2-2bPTIII.	
R2-2bPTIV-P350	The 350 bp PDS fragment was inserted in reverse orientation between the BamHI and SmaI restriction sites of R2-2bPTIV.	
R2-2bPTIV-P400	The 400 bp PDS fragment was inserted in reverse orientation between the BamHI and SmaI restriction sites of R2-2bPTIV.	
R2-2bPTIV-P450	The 450 bp PDS fragment was inserted in reverse orientation between the BamHI and SmaI restriction sites of R2-2bPTIV.	Assessment Ability of R2-2bPTIV to accommodate exogenous fragments
R2-2bPTIV-P500	The 500 bp PDS fragment was inserted in reverse orientation between the BamHI and SmaI restriction sites of R2-2bPTIV.	
R2-2bPTIV-P550	The 550 bp PDS fragment was inserted in reverse orientation between the BamHI and SmaI restriction sites of R2-2bPTIV.	
R2-2bPTIV-P600	The 600 bp PDS fragment was inserted in reverse orientation between the BamHI and SmaI restriction sites of R2-2bPTIV.	
R2-2bPTIV-P800	The 800 bp PDS fragment was inserted in reverse orientation between the BamHI and SmaI restriction sites of R2-2bPTIV.	
R2-2bPTV-P450	The 450 bp PDS fragment was inserted in reverse orientation between the BamHI and SmaI restriction sites of R2-2bPTV.	
R2-2bPTV-P500	The 500 bp PDS fragment was inserted in reverse orientation between the BamHI and SmaI restriction sites of R2-2bPTV.	Assessment Ability of R2-2bPTV to accommodate exogenous fragments
R2-2bPTV-P550	The 550 bp PDS fragment was inserted in reverse orientation between the BamHI and SmaI restriction sites of R2-2bPTV.	
R2-2bPTV-P600	The 600 bp PDS fragment was inserted in reverse orientation between the BamHI and SmaI restriction sites of R2-2bPTV.	
R2-2bPTV-P800	The 800 bp PDS fragment was inserted in reverse orientation between the BamHI and SmaI restriction sites of R2-2bPTV.	

Name	Mutant site information	Purpose
R2-2bPTVI-P450	The 450 bp PDS fragment was inserted in reverse orientation between the BamHI and SmaI restriction sites of R2-2bPTVI.	Assessment<br>Ability of R2-2bPTVI to accommodate exogenous fragments
R2-2bPTVI-P500	The 500 bp PDS fragment was inserted in reverse orientation between the BamHI and SmaI restriction sites of R2-2bPTVI.	
R2-2bPTVI-P550	The 550 bp PDS fragment was inserted in reverse orientation between the BamHI and SmaI restriction sites of R2-2bPTVI.	
R2-2bPTVI-P600	The 600 bp PDS fragment was inserted in reverse orientation between the BamHI and SmaI restriction sites of R2-2bPTVI.	
R2-2bPTVI-P800	The 800 bp PDS fragment was inserted in reverse orientation between the BamHI and SmaI restriction sites of R2-2bPTVI.	
R2-2bPTIV-$T_MP_YT_VP_X$	The 121bp TMV, 100bp PVY, 100bp TVBMV and 100bp PVX fragments were inserted between the BamHI and SmaI restriction sites of R2-2bPTIV.	Cross-protection Application accessment of<br>R2-2bPTIV

Note: enzyme sites: underlined; stop codon: bold
